# The Changing Pattern of Population Structure of *Staphylococcus aureus* from Bacteremia in China from 2013 to 2016: ST239-030-MRSA Replaced by ST59-t437

**DOI:** 10.3389/fmicb.2018.00332

**Published:** 2018-02-27

**Authors:** Shuguang Li, Shijun Sun, Chentao Yang, Hongbin Chen, Yuyao Yin, Henan Li, Chunjiang Zhao, Hui Wang

**Affiliations:** ^1^Department of Clinical Laboratory, Peking University People's Hospital, Beijing, China; ^2^Department of Pharmacology, Xingtai Medical College, Xingtai, China

**Keywords:** MRSA, bacteremia, population structure, genotype, fitness cost, *in vitro* competition

## Abstract

To investigate the epidemiology and genetic structure of *Staphylococcus aureus* bacteremia in China, a total of 416 isolates from 22 teaching hospitals in 12 cities from 2013 and 2016 were characterized by antibiogram analysis, multilocus sequence typing (MLST), *spa* typing and staphylococcal cassette chromosome *mec* (SCC*mec*) typing. The predominant meticillin-susceptible (MSSA) genotypes in 2013 were ST188 (19.1%), ST7 (8.7%), and ST398 (7.8%), respectively, and they continued to be the main genotypes in 2016. The prevalence of meticillin-resistant *S. aureus* (MRSA) were 36.5% (66/181) and 36.6% (86/235) in 2013 and 2016, respectively. Interestingly, the susceptibility rates of MRSA to rifampicin and fluoroquinolones increased significantly from 2013 to 2016 (*P* < 0.01), and this was associated with changes in genetic structure. ST239-t030-MRSA, the predominant genotype among all MRSAs in 2013 (34.8%), was replaced by ST59-t437-MRSA (15.1%) in 2016. Further analysis revealed that the ST239-t030-MRSA were more resistant to rifampicin, tetracycline and fluoroquinolones than ST59-t437-MRSA (*P* < 0.01). To further gain insight into the mechanisms underlying the changes of genetic structure, *in vitro* competition and fitness measurements were performed. Importantly, ST239-t030-MRSA displayed lower growth rate and lower competitive advantage compared to ST59-t437-MRSA. Together, our findings reveal that fitness advantage of ST59-t437-MRSA over ST239-t030-MRSA may lead to changes in genetic structure and increased susceptibility of MRSA to rifampicin and fluoroquinolones in Chinese patients with *S. aureus* bacteremia. Our study supports temporal dynamics in MRSA clone diversities, further providing critical insights into the importance of continued monitoring of MRSA.

## Introduction

Gram positive *Staphylococcus aureus* is one of the most common pathogens of hospital- and community-acquired infections with substantial morbidity and mortality (Li et al., 2016; Musicha et al., 2017). The mortality rate associated with serious *S. aureus* infection, especially bacteremia, is as high as 15–25% (Vogel et al., 2016; Asgeirsson et al., 2017; Recker et al., 2017). The *S. aureus* bacteremia is further exacerbated by the emergence and widespread circulation of drug-resistant strains, especially methicillin-resistant *S. aureus* (MRSA) (Gordon and Lowy, 2008; Recker et al., 2017).

The multicenter surveillance network is important in monitoring bacterial resistance in different regions and providing crucial insight into clinical infection control and rational use of antibiotics. One of the important tools in bacterial resistance surveillance is to track population structure changes for a given pathogen. In *S. aureus*, staphylococcal cassette chromosome *mec* (SCC*mec*) element, multilocus sequence typing (MLST), and *spa* typing have been widely used (Ito et al., 1999; Enright et al., 2000; Harmsen et al., 2003). Utilizing those methods, we have previously shown that ST239-MRSA-SCC*mec*III and ST5-MRSA-SCC*mec*II were the predominant clones in Chinese hospitals in 2005–2006 and 2010–2011, respectively, while ST7-t091/t796, ST188-t189, and ST398-t571/t034 were the most prevalent methicillin-sensitive *S. aureus* (MSSA) bacteremia clones (Liu et al., 2009; He et al., 2013). This study aimed at investigating whether the population structure of *S. aureus* causing bacteremia has changed over the past several years and determine the underlying mechanism leading to the temporal dynamics in MRSA clone diversities.

## Materials and methods

### Bacterial isolates collection

A total of 416 consecutive, non-duplicate *S. aureus* bacteremia isolates were collected from 22 teaching hospitals in 12 cities from January–December in 2013 (181 isolates) and 2016 (235 isolates); the cities including Beijing, Changsha, Chongqing, Guangzhou, Hangzhou, Jinan, Nanjing, Shanghai, Shenyang, Tianjin, Wuhan and Xi'an, representing the capitals of 12 different provinces, and were distributed in distant geographic areas in China (the surveyed cities were same as that referred in He et al., 2013). These isolates were provided by the Gram Positive Cocci Resistance Surveillance (GPRS) networks and Chinese Antimicrobial Resistance Surveillance of Nosocomial Infections (CARES) networks (detail information of these two networks was shown in Acknowledgments). For GPRS networks, each center was required to send 50 non-repetitive consecutive Gram-positive cocci isolates in 2013, and 20 non-repetitive consecutive bacteremia *S. aureus* isolates in 2016; for CARES networks, 180 non-repetitive consecutive isolates from bloodstream infection, hospital-acquired pneumonia and intra-abdominal infection were sent from each center in 2013 and 2016. *S. aureus* isolates were re-identified by routine microbiology/biochemical methods, and/or the Vitek® 2 system (bioMérieux, Hazelwood, MO, US) in Peking University People's Hospital (the Central Laboratory). All isolates were stored at −80°C. The study was approved by the research ethics board at Peking University People's Hospital. Informed consent was not needed as this was a retrospective study and patients were anonymized.

### Antimicrobial susceptibility testing

The minimum inhibitory concentrations (MICs) of routine clinical antibiotics were determined using broth microdilution method (tedizolid and daptomycin) or agar dilution method (agents other than tedizolid and daptomycin) according to the CLSI guidelines (M7-A9) (CLSI, 2012). Isolates were categorized into resistant (R), intermediate (I), or susceptible (S) according to CLSI M100-S27 (CLSI, 2017). The MIC interpretive criteria for tigecycline were as recommended by the US Food and Drug Administration (FDA). *S. aureus* ATCC 29213 was used as quality control.

### Molecular typing methods

All *S. aureus* isolates were investigated by MLST and *spa* typing. Genomic DNA was prepared using DNA Extraction Kit (TIANGEN Biotech, China). MLST was performed as described previously and the sequences of the PCR products were compared with an MLST database (http://saureus.mlst.net) (Enright et al., 2000); a neighbor-joining tree was constructed with the concatenated sequences of the seven MLST genes (*arcc, aroe, glpf*, *gmk, pta, tpi*, and *yqil*) using MEGA5 (Tamura et al., 2011), and only one sequence from the same sequence types (STs) was used to avoid duplication. The clustering of STs was performed using eBURST V3 algorithm (http://eburst.mlst.net/) by comparing the present dataset to that of the *S. aureus* MLST database of 4408 STs by the end of November 2017 (https://pubmlst.org/saureus/), and ran with the default settings. STs that showed at least six of seven identical alleles were grouped into clonal complexes (CC). The *spa* genes were sequenced and *spa* types were assigned based on comparisons with the *spa* database (http://spa.ridom.de/spatypes.shtml) (Harmsen et al., 2003). Genes encoding the cassette chromosome recombinase (*ccr*) complex and *mec* complex were typed by multiplex PCR as previously described (Zhang et al., 2009, 2012).

### Growth assay and calculation of generation times

The randomly selected four ST59-t437-MRSA isolates, 2016-ST59-t437-1, 2016-ST59-t437-2, 2013-ST59-t437-1, 2013-ST59-t437-2, and four ST239-t030-MRSA isolates, 2016-ST239-t030-1, 2016-ST239-t030-2, 2013-ST239-t030-1, and 2013-ST239-t030-2, were shortly named A_1_, A_2_, B_1_, B_2_, C_1_, C_2_, D_1_, D_2_, respectively. Isolates were cultured overnight in LB broth, diluted to an OD_600_ of 0.01 and grown at 37°C with agitation at 200 rpm. The cell density was determined every 0.5 h by measuring the OD_600_. All these eight randomly selected *S. aureus* isolates were cultured to logarithmic growth phase (OD_600_ = 0.3) in LB broth at 37°C with agitation at 200 rpm. The numbers of colonies were counted at the beginning and after 60 min of time interval. Then the generation time was calculated as

(1) $$\begin{array}{r} \text{G}=\frac{t}{3.3\times lg\frac{b}{B}}, \end{array}$$

where G = generation time, *t* = time interval, *b* = number of bacteria at the end of the time interval, and *B* = number of bacteria at the beginning of a time interval (Smits and Riemann, 1988; Li et al., 2017).

### *In vitro* competition and fitness measurements

The randomly selected eight isolates were diluted to 0.5 × 10^7^ colony-forming units (CFU)/ml, equal volumes were combined, thus the initial ratio of the isolate pairs was close to 1:1, then 10 μl of the mixture was added to 20 ml LB broth and cultured at 37°C with agitation at 200 rpm. At 24-h intervals, 10 μl bacterial subcultures were transferred to fresh LB broth; meanwhile, 10 μl was inoculated on drug-free MH agar, and 10 μl on MH agar containing 1 μg/ml rifampicin. The numbers of ST59-t437 (susceptible to rifampicin, MIC ≤ 0.016 μg/ml) and ST239-t030 (resistant to rifampicin, MIC ≥ 128 μg/ml) colonies were counted, and after 96 h, adaptive difference was calculated as

(2) $$\begin{array}{r} S=\ln\left[{\left(\frac{\frac{r_{t}}{S_{t}}}{\frac{r_{t-1}}{S_{t-1}}}\right)}^{\left(\frac{1}{17}\right)}\right], \end{array}$$

relative adaptive fitness as

(3) $$\begin{array}{r} F=1+S, \end{array}$$

and the fitness cost as

(4) $$\begin{array}{r} \text{C}=\left(1-F\right)\times 100\text{\%}, \end{array}$$

where *r*_*t*_ = number of resistant colonies and *s*_*t*_ = number of sensitive colonies (Sander et al., 2002; Guo et al., 2012; Nielsen et al., 2012; Li et al., 2017).

### Statistical analysis

All susceptibility data and molecular test results were analyzed using the software WHONET-5.6. Statistical analysis of growth curves were performed with the software GraphPad Prism version 5 using one-way analysis of variance (ANOVA) followed by Tukey–Kramer tests; percentage values or frequencies were analyzed pairwise by two tailed chi-square test or Fisher's exact test, if appropriate. *P* < 0.05 was considered to be statistically significant.

## Results

### The prevalence of MRSA

Among the 416 bacteremia *S. aureus* isolates obtained in 2013 (*n* = 181) and 2016 (*n* = 235), the average prevalence of MRSA were 36.5% (66/181) and 36.6% (86/235), respectively. The distribution of MRSA from the 12 investigative cities is shown in Table 1. For cities where the number of *S. aureus* isolates was more than 10, the prevalence of MRSA was calculated. The prevalence of MRSA ranged from 18.8% (6/32) in Guangzhou to 55.5% (11/20) in Wuhan in 2013; and from 27.3% (15/55) in Beijing to 60.0% (12/20) in Shanghai in 2016.

**Table 1:** Distribution of meticillin-resistant *Staphylococcus aureus* (MRSA) from bacteremia in 12 cities throughout China in 2013 and 2016.

**City**	**2013**		**2016**	
	**No. of isolates**	No. of MRSA isolates (% MRSA)^*^	**No. of isolates**	No. of MRSA isolates (% MRSA)^*^
Beijing	26	6 (23.1%)	55	15 (27.3%)
Changsha	7	1	7	1
Chongqing	3	1	19	6 (31.6%)
Guangzhou	32	6 (18.8%)	10	3 (30.0%)
Hangzhou	13	5 (38.5%)	47	19 (40.4%)
Jinan	6	3	4	0
Nanjing	9	3	20	9 (45.0%)
Shanghai	21	9 (42.9%)	20	12 (60.0%)
Shenyang	19	10 (52.6%)	9	1
Tianjin	8	2	9	2
Wuhan	20	11 (55.0%)	23	11 (47.8%)
Xi'an	17	9 (52.9%)	12	7 (58.3%)
Total	181	66 (36.5%)	235	86 (36.6%)

* *The prevalence of MRSA was not calculated in cities where the number of S. aureus was < 10*.

### Antimicrobial activities

All isolates were susceptible to vancomycin, teicoplanin, tigecycline, linezolid, tedizolid, and daptomycin. The MIC_50_, MIC_90_ and geometric mean MICs of those antimicrobials remained similar between 2013 and 2016 (Table 2). In addition to the above-mentioned antimicrobials, trimethoprim-sulfamethoxazole displayed the best antimicrobial activity (93.9–98.8% sensitivity in MRSA and 98.3–99.3% sensitivity in MSSA), while erythromycin exhibited the poorest antimicrobial activity (9.3–10.6% sensitivity in MRSA and 52.3–60.0% sensitivity in MSSA). Interestingly, the susceptibility rates of MRSA to rifampicin and fluoroquinolones (moxifloxacin, levofloxacin, and ciprofloxacin) were significantly increased from 2013 to 2016 (20.8–28.4% of increase, *P* < 0.01). The antimicrobial resistant profiles among all MSSAs remained similar from 2013 to 2016 (data not shown).

**Table 2:** Activity profile of antibiotics against all meticillin-resistant *Staphylococcus aureus* (MRSA) isolates collected in China in 2013 and 2016 (MIC, μg/ml).

**Drugs**	**2013 (*n* = 66)**					**2016 (*n* = 86)**				
	**%S**	**MIC_50_**	**MIC_90_**	**Geometric mean**	**MIC range**	**%S**	**MIC_50_**	**MIC_90_**	**Geometric mean**	**MIC range**
VAN	100	1	1	0.872	0.5–1	100	1	1	0.785	0.5–2
TEC	100	1	2	1.122	0.25–4	100	1	4	1.243	0.25–8
TGC	100	0.25	0.5	0.272	0.125–0.5	100	0.25	0.5	0.337	0.125–0.5
LNZ	100	2	2	1.429	1–2	100	1	2	1.213	1–4
TZD	100	0.25	0.25	0.218	0.125–0.25	100	0.5	0.5	0.392	0.25–0.5
DAP	100	0.5	0.5	0.418	0.125–1	100	0.5	1	0.551	0.125–1
SXT	93.9	0.125	0.5	0.116	0.016–64	98.8	0.032	0.125	0.055	0.016–16
CHL	89.4	8	32	8.431	4–128	89.5	8	16	6.920	4 −64
RIF^*^	57.6	0.016	256	0.619	0.004–512	86.0	0.012	256	0.036	0.004–256
MFX^*^	19.7	8	8	2.557	0.016–128	40.7	4	8	0.921	0.016–64
LVX^*^	19.7	32	64	12.435	0.125–128	40.7	16	32	4.336	0.125–256
CIP^*^	15.2	64	128	28.221	0.004–128	36.0	64	128	11.874	0.25–128
TCY	20	64	64	18.700	0.125–128	35.1	16	64	6.514	0.032–128
CLI	40.9	>256	>256	14.197	0.064–512	34.9	256	>256	16.377	0.016–512
ERY	10.6	>256	>256	96.397	0.125–512	9.3	>256	>256	112.513	0.25–512

*VAN, vancomycin; TEC, teicoplanin; TGC, tigecycline; LNZ, linezolid; TZD, tedizolid; DAP, daptomycin; SXT, trimethoprim–sulfamethoxazole; CHL, chloramphenicol; RIF, rifampin; MFX, moxifloxacin; LVX, levofloxacin; CIP, ciprofloxacin; TCY, tetracycline; CLI, clindamycin; ERY, erythromycin; S%, percentage of susceptibility rates*.

* *The susceptibility rates of MRSA to these four drugs (RIF, MFX, LVX, and CIP) in 2016 increased significantly over 2013 (P < 0.01)*.

### Molecular typing and prevalence of genotypes

**Figure 1:**
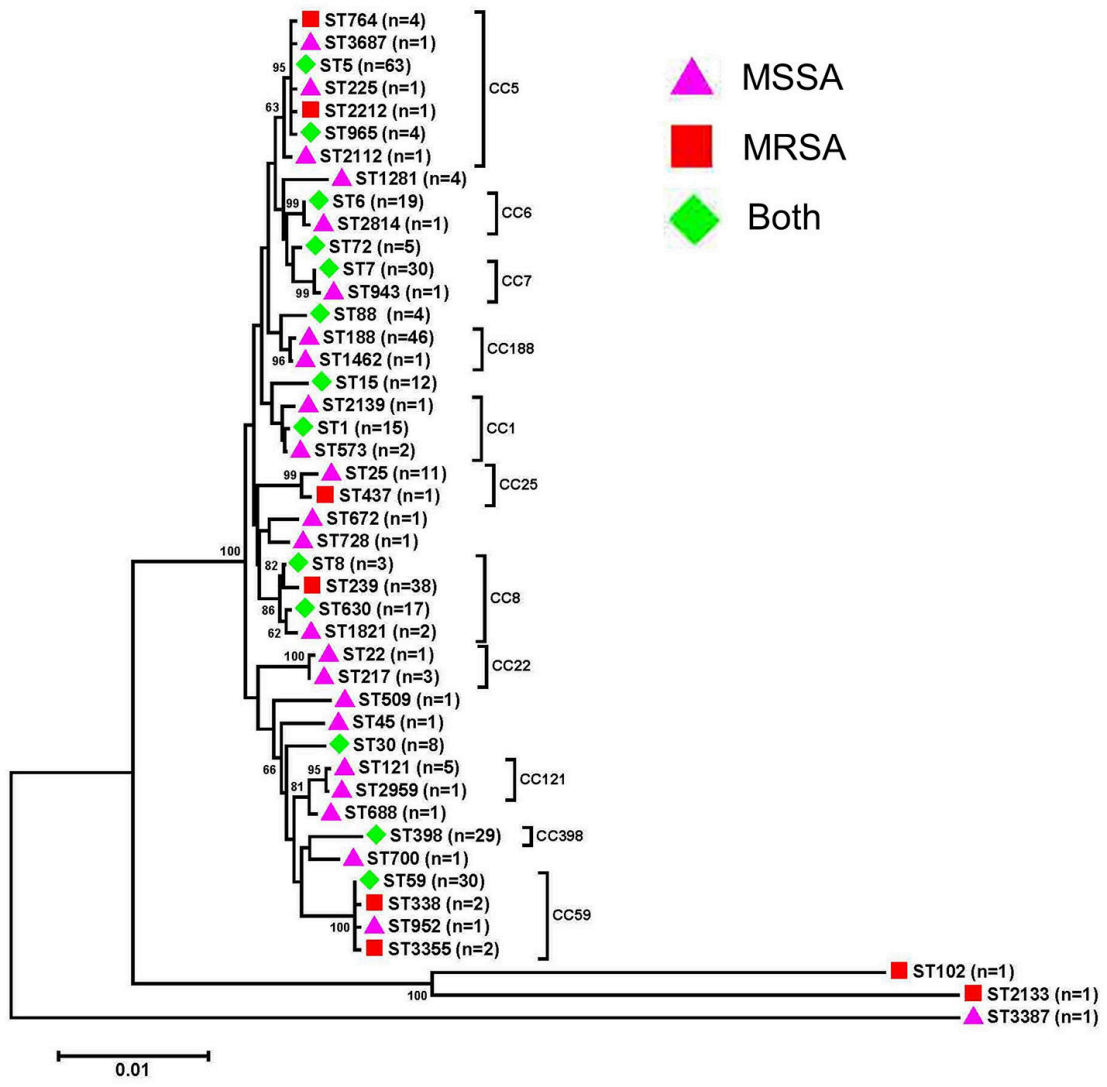
Phylogenetic analysis of multilocus sequence types (STs) of bacteremia *Staphylococcus aureus* isolates. The neighbor-joining tree was constructed with the concatenated sequences of the seven MLST genes (*arcc, aroe, glpf, gmk, pta, tpi*, and *yqil*) based on the distance matrix of pair-wise differences between STs. Bootstrap support was based on 1,000 replicates, and only branch nodes higher than 60 were shown. The bar is equivalent to one nucleotide change per 100 bp. MSSA, meticillin-susceptible *S. aureus*; MRSA, meticillin-resistant *S. aureus*.

A total of 45 STs, which belonged to 25 CCs, and 111 *spa* types were identified in 2013 (28 STs and 61 *spa* types) and 2016 (32 STs and 82 *spa* types) (Figure 1 and Table 3). CC5, CC8, CC188, CC59, CC7, and CC398 were the most prevalent CCs, including 18.0% (75/416), 14.4% (60/416), 11.3% (47/416), 8.4% (35/416), 7.5% (31/416), and 7.0% (29/416) of the isolates, respectively. An MLST dendrogram indicated that ST1, ST5, ST6, ST7, ST8, ST15, ST30, ST59, ST72, ST88, ST398, ST630, and ST965 included both MRSA and MSSA isolates (Figure 1). ST239 genotypes were all MRSA isolates. For ST398, ST6, ST630, and ST1, all the isolates were MSSA in 2013; while MRSA were present in 25% (5/20), 8.3% (1/12), 36.4% (4/11), and 50.0% (1/2) of those isolates in 2016, respectively.

**Table 3:** The 11 predominant multilocus sequence types (MLSTs) and relationship between various molecular types of 416 *Staphylococcus aureus* bacteremia isolates in 2013 and 2016.

**CC**	**2013 MRSA (*n* = 66)**			**2013 MSSA (*n* = 115)**		**2016 MRSA (*n* = 86)**			**2016 MSSA (*n* = 149)**		**Total**
	**MLST (no. of isolates)**	**SCC*mec* (no. of isolates)**	***spa* types (no. of isolates)**	**MLST (no. of isolates)**	***spa* types (no. of isolates)**	**MLST (no. of isolates)**	**SCC*mec* (no. of isolates)**	***spa* types (no. of isolates)**	**MLST (no. of isolates)**	***spa* types (no. of isolates)**	
CC5	ST5 (14)	II (13); III (1)	t311 (5); t570 (3); t002 (2); t2460 (2); t062 (1); t319 (1)	ST5 (7)	t002 (4); t062 (1); t179 (1); t548 (1)	ST5 (27)	II (27)	t2460 (8); t311 (7); t586 (5); t002 (2); t085 (1); t264 (1); t548 (1); t796 (1); NA (1)	ST5 (15)	t002 (7); t548 (3); t214 (1); t954 (1); t13142 (1); t2460 (1); t5353 (1)	63
CC188				ST188 (22)	t189 (22)				ST188 (24)	t189 (16); t2152 (1); t2174 (1); t2445 (1); t2765 (1); t3019 (1); t4209 (1); t4445 (1); NA (1)	46
CC8	ST239 (28)	III (28)	t030 (23); t037 (2); t459 (2); t233 (1)			ST239 (10)	III (9); V (1)	t030 (9); t037 (1)			38
CC59	ST59 (6)	IV (5); III (1)	t437 (6)	ST59 (5)	t437 (4); t163 (1)	ST59 (17)	IV (10); I (5);III (1); V(1)	t437 (13); t163 (2); t3590 (1); t12011 (1)	ST59 (2)	t437 (2)	30
CC7	ST7(2)	NA	t091 (2)	ST7 (10)	t091 (5); t796 (3); t289 (2)				ST7 (18)	t796 (9); t091 (7); t1943 (1); t2460 (1)	30
CC398				ST398 (9)	t571 (5); t1451 (2); t011 (1); t034 (1)	ST398 (5)	NA	t034 (4); t011 (1)	ST398 (15)	t034 (7); t571 (2); t1451 (2); t1250 (1); t1928 (1); t3615 (1); t6587 (1)	29
CC6				ST6 (7)	t701 (4); t2915 (2); t304 (1)	ST6 (1)	II (1)	t8657 (1)	ST6 (11)	t701 (6); t304 (1); t1381 (1); t2915 (1); t5268 (1); t8657 (1)	19
CC8				ST630 (6)	t377 (5); t3386 (1)	ST630 (4)	II (2); V (2)	t4549 (2); t8410 (1); t11041 (1)	ST630 (7)	t377 (4); t5773 (1); t8410 (1); t11041 (1)	17
CC1				ST1 (8)	t127 (6); t286 (2)	ST1 (1)	NA	NA (1)	ST1 (6)	t127 (4); t177 (1); t1381 (1)	15
CC15	ST15 (1)	NA	t084 (1)	ST15 (5)	t084 (2); t085 (1); t803 (1); t346 (1)				ST15 (6)	t084 (1); t144 (1); t547 (1); t803 (1); t14014 (1); NA (1)	12
CC25				ST25 (3)	t078 (3)				ST25 (8)	t3292 (5); t078 (2); t11793 (1)	11
	Others (15)			Others (33)		Others (21)			Others (37)		

*MRSA, meticillin-resistant S. aureus; MSSA, meticillin-susceptible S. aureus; SCCmec, staphylococcal cassette chromosome mec; NA, not available*.

The 11 predominant STs containing isolates more than 10 were shown in detail in Table 3. The six predominant STs (ST5, ST188, ST239, ST59, ST7, and ST398) included 15.1% (63/416), 11.1% (46/416), 9.1% (38/416), 7.2% (30/416), 7.2% (30/416), 7.0% (29/416) of the isolates. When ST types and oxacillin (OXA) sensitivity were combined, the six predominant genotypes in 2013 were ST239-MRSA (15.5%), ST188-MSSA (12.2%), ST5-MRSA (7.7%), ST7-MSSA (5.5%), ST398-MSSA (5.0%), and ST1-MSSA (4.4%), while in 2016 were ST5-MRSA (11.5%), ST188-MSSA (10.2%), ST7-MSSA (7.7%), ST59-MRSA (7.2%), ST5-MSSA (6.4%), and ST398-MSSA (6.4%). Notably, the prevalence of ST239-MRSA was significantly decreased from 2013 to 2016 (15.5% vs. 4.3%, *P* < 0.001). Instead, ST5-MRSA and ST59-MRSA became the two predominant MRSA genotypes in 2016. ST188-MSSA and ST7-MSSA were the two predominant MSSA genotypes in both 2013 and 2016. When ST types, *spa* types and OXA sensitivity were combined analysis, 72 and 119 genotypes were obtained in 2013 and 2016, respectively; the ratio of the number of genotypes to the number of isolates was significantly increased from 2013 to 2016 (0.398 vs. 0.506, *P* < 0.05).

### Activity profile of antibiotics to ST59-t437 and ST239-t030 MRSA isolates

All ST239-t030 were MRSA isolates (*n* = 32); while the prevalence of MRSA in ST59-t437 were 60% (6/10) in 2013 and 86.7% (13/15) in 2016. Importantly, ST239-t030, the predominant genotype among all MRSAs in 2013 (34.8%), was replaced by ST59-t437 (15.1%) in 2016. The susceptibility rates of ST59-t437-MRSA (*n* = 19) to rifampicin (100%), moxifloxacin (94.7%), levofloxacin (94.7%), ciprofloxacin (78.9%), and tetracycline (46.2%) were all significantly higher than those from ST239-t030-MRSA (*n* = 32, all the isolates were resistant to those antimicrobials, *P* < 0.01). In addition, the predominant resistance profile of ST239-t030-MRSA was oxacillin-ciprofloxacin-levofloxacin-moxifloxacin-erythromycin-clindamycin-tetracycline-rifampin in 2013 (30.4%, 7/23) and 2016 (55.5%, 5/9); while the predominant resistance profile of ST59-t437-MRSA was oxacillin-erythromycin-clindamycin-tetracycline in 2013 (16.7%, 1/6) and 2016 (61.5%, 8/13).

### Growth rates of ST59-t437 and ST239-t030 MRSA isolates

**Figure 2:**
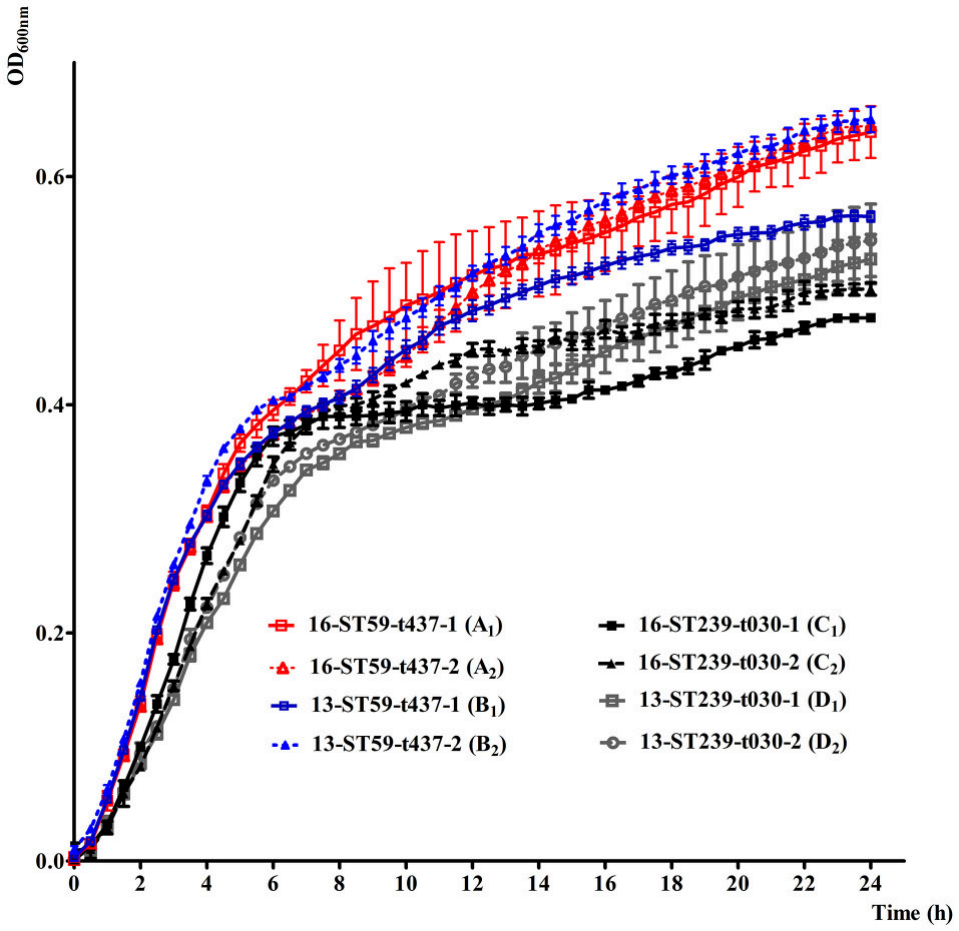
*In vitro* growth characteristics of random selected ST59-t437 and ST239-t030 meticillin-resistant *Staphylococcus aureus* (MRSA) isolates cultured at 37°C in LB broth. The means and standard deviation of three independent experiments are shown. The abbreviated codes (A_1_, A_2_, B_1_, B_2_, C_1_, C_2_, D_1_ and D_2_) for the isolates are listed in the brackets. Among the pairwise comparison of growth curves, A_1_C_1_, B_2_C_1_, and B_2_D_1_ showed significant statistical differences (*P* < 0.05), when using one-way analysis of variance (ANOVA) followed by Tukey–Kramer tests.

To further gain insight into the mechanisms underlying the changes of genetic structure, growth rates between ST59-t437 and ST239-t030 were examined. Growth characteristics of the randomly selected ST59-t437 and ST239-t030 MRSA isolates are shown in Figure 2. The OD_600_ values of the four ST59-t437 isolates were higher than that of the ST239-t030 isolates throughout the 24 h. Among the pairwise comparison of growth curves, A_1_C_1_, B_2_C_1_, and B_2_D_1_ showed significant statistical differences (*P* < 0.05), i.e., the growth of A_1_ was significantly faster than C_1_; B_2_ was significantly higher than C_1_ and D_1_. The average generation time of ST59-t437 (A_1_, 40 min; A_2_, 25 min; B_1_, 27 min; B_2_, 25 min) and ST239-t030 isolates (C_1_, 38 min; C_2_, 33 min; D_1_, 33 min; D_2_, 42 min) were 29 ± 6 (min) and 37 ± 3 (min), respectively. Taken together, ST59-t437 isolates demonstrated faster growth rates compared to ST239-t030 isolates.

### *In vitro* competition between ST59-t437 and ST239-t030 MRSA isolates

*In vitro* competition experiments showed that ST59-t437 exhibited competitive advantage in three pairs (A_1_C_2_, A_1_D_1_, and A_1_D_2_) with the fitness cost *C* > 10%. In addition, a fourth pair (A_2_D_1_) displayed slightly advantage with C-value of 3.8% (Table 4). ST59-t437 and ST239-t030 showed similar fitness in the rest 4 pairs (the absolute *C* < 1.5%). Thus, ST59-t437 exhibited a trend toward better fitness compared to ST239-t030.

**Table 4:** *In vitro* competition results between ST59-t437 and ST239-t030 meticillin-resistant *Staphylococcus aureus* isolates.

**Control strain**	**Experimental strain**	**Relative adaptive fitness (F) (%)**	**Fitness cost (C value) (%)**
2016-t437-1 (A_1_)	2016-t030-1 (C_1_)	98.9	1.1
2016-t437-1 (A_1_)	2016-t030-2 (C_2_)	88.9	11.1
2016-t437-1 (A_1_)	2013-t030-1 (D_1_)	89.6	10.4
2016-t437-1 (A_1_)	2013-t030-2 (D_2_)	88.6	11.4
2016-t437-2 (A_2_)	2016-t030-1 (C_1_)	101.4	−1.4
2016-t437-2 (A_2_)	2013-t030-1 (D_1_)	96.2	3.8
2013-t437-1 (B_1_)	2016-t030-1 (C_1_)	100.4	−0.4
2013-t437-1 (B_1_)	2013-t030-1 (D_1_)	99.3	0.7

*The abbreviated codes (A_1_, A_2_, B_1_, B_2_, C_1_, C_2_, D_1_, and D_2_) for the isolates are listed in the brackets*.

## Discussion

This study aimed at investigating whether the population structure of *S. aureus* causing bacteremia has changed over the past several years and determine the underlying mechanism. The prevalence of MRSA were 36.5% and 36.6% in 2013 and 2016, respectively, which were lower than those from our previous study in 2010–2011 (47.5%) (He et al., 2013), and were lower than those from Latin America in 2011–2014 (45.4%) (Arias et al., 2017). Interestingly, Shanghai (42.9–60.0%) and Xi'an (52.9–58.3%) continued to show high MRSA prevalence from 2010 to 2016 (He et al., 2013). The prevalence of MRSA in Beijing was declined from 36.7% to 23.1–27.3%. In contrast, the prevalence of MRSA from Wuhan was increased from 31.5% in 2010–2011 to 47.8–55.0% in 2013 and 2016. Of note, the prevalence of MRSA decreased in most cities in China since 2011, which is similar to a previous study from Finland (Jokinen et al., 2017). Interestingly, the susceptibility rates of MRSA to fluoroquinolones were significantly increased from 2013 to 2016, which is also a trend recently reported in MRSA from Germany (Schaumburg et al., 2014).

One of the most important findings in this study is the population structure change among MRSA isolates. The prevalence of ST239-MRSA was significantly decreased from 2013 (42.4%) to 2016 (11.6%) (*P* < 0.001). Multidrug-resistant ST239 is one of the most successful and persistent clones that globally dispersed (Baines et al., 2015), and was the most predominant genotype in our previous studies (Liu et al., 2009; He et al., 2013). ST239 had two important *spa* types, t030, and t037. ST239-t030-MRSA, the most predominant genotype in China from 2005 to 2013, decreased significantly in 2016 (10.5%) (*P* < 0.001) (Liu et al., 2009; He et al., 2013). This genotype was mainly distributed at Wuhan (39.1%) and Beijing (33.3%) in 2013 and 2016, respectively. Of note, ST239-t030-MRSA isolates were all resistant to rifampicin, tetracycline and fluoroquinolones (moxifloxacin, levofloxacin and ciprofloxacin). The resistance genes they carry or acquire may be a burden in evolution (Baines et al., 2015; Lee et al., 2015; Li et al., 2017). Similarly, the prevalence of ST239-t037-MRSA was decreased from 2010 to 2016 (13.4% in 2010–2011 vs. 3.0% in 2013 and 1.2% in 2016).

ST5 contained both MRSA and MSSA isolates, and MRSA prevalence in this ST was slightly reduced from 2010–2011 to 2016 (73.7% vs. 64.3%; He et al., 2013). Interestingly, the prevalence of ST5 was increased among all STs in MRSA from 2010–2011 to 2016 (12.5–31.4%). In addition, we noticed that the *spa* profiles in this ST has changed during the past seven years: t570 was the predominant one in 2010–2011, while t311 became the main genotype in 2013 and t2460 became the main genotype in 2016. The prevalence of MRSA within ST59 were 54.5% and 89.5% in 2013 and 2016, respectively. Four *spa* types were obtained in this study, t437 (r04-r20-r17-r20-r17-r25-r34), t163 (r04-r20-r17-r20-r17-r45-r16-r34), t3590 (r04-r02-r17-r20-r17-r25-r34) and t12011 (r04-r02-r17-r20-r17-r25-r34-r34). Taken the repeat pattern into consideration, these four *spa* types might be originate from two ancestors. Importantly, the prevalence of ST59-t437-MRSA among all MRSAs increased from 3.6% in 2010–2011 (He et al., 2013) to 9.1% in 2013, and there was a further increase in 2016 (15.1%). The geographical distribution of ST59-t437-MRSA was scattered, while Guangzhou (2/6) and Chongqing (3/13) were the major distributed cities in 2013 and 2016, respectively.

As ST239-t030-MRSA displayed more resistant rates to multiple antimicrobials compared to ST59-t437-MRSA, we investigated whether the presence of multi-drug resistant genes represents the “fitness cost,” making them less competitive (Baines et al., 2015; Lee et al., 2015; Li et al., 2017). Indeed, ST239-t030-MRSA displayed lower growth rate and a trend toward lower competitive advantage compared to ST59-t437-MRSA. Thus, the fitness cost may lead to the replacement of ST239-t030-MRSA by ST59-t437-MRSA, which correlated with the increased susceptibility of MRSA population to rifampicin and fluoroquinolones.

The present study showed that ST5 and ST59 were the most predominant bacteremia MRSA genotypes in China in 2016, which is different from findings in other countries: the majority of bacteremia MRSA isolates were ST5 and ST8 in Latin America in 2011–2014 and Minnesota, US in 2015 (Arias et al., 2017; Park et al., 2017), ST22 and ST239 in Australian in 2014 (Coombs et al., 2016), ST239 and ST22 in Iran in 2015–2016 (Goudarzi et al., 2017). Even in China, ST239 was still the predominant genotype in a burn center of Chongqing in 2011–2016 (Jiang et al., 2017). These studies complement our understandings of population structure of bacteremia *S. aureus*.

The predominant MSSA genotypes have remained basically unchanged: ST188-MSSA (13.7–19.1%), ST7-MSSA (8.7–18.5%), and ST398-MSSA (7.3–10.1%) were the three predominant MSSA genotypes from 2010–2011 to 2016. Interestingly, ST398 contained only MSSA in 2010–2011 and 2013, but MRSA strains occurred in Beijing (*n* = 2), Nanjing (*n* = 2) and Shanghai (*n* = 1) in 2016, which correlated with patients with infective endocarditis, nephropathy or hepatic encephalopathy. The emergence of ST398-t034/t011-MRSA might be due to an import of this clone from Europe, where livestock-associated MRSA (LA-MRSA) is widespread, and an increase of LA-MRSA has been reported for some regions in Europe (Schaumburg et al., 2012). However, these five ST398-MRSA isolates were susceptible to all surveyed drugs except erythromycin (two isolates were resistant to erythromycin). The occurrence of ST398-MRSA in China is important, which should be taken seriously and further studied with whole genome sequencing.

The limitations of present study were that the number of selected isolates for competition experiments was small, may not reflect the whole genotype population, and no genomic information supported for the growth advantage of ST59-t437-MRSA isolates. Further research would increase the sample size and do complete genomic comparison.

In conclusion, our findings reveal that fitness advantage of ST59-t437-MRSA over ST239-t030-MRSA may lead to changes in genetic structure and increased susceptibility of MRSA to rifampicin and fluoroquinolones in Chinese patients with *S. aureus* bacteremia. Our study supports temporal dynamics in MRSA clone diversities, further providing critical insights into the importance of continued monitoring of MRSA.

## Author contributions

HW conceived and designed the study. SL, SS, CY, HC, YY, HL, and CZ performed experiments described in this study. SL wrote the draft, and HW revised it. All authors approved the final version.

### Conflict of interest statement

The authors declare that the research was conducted in the absence of any commercial or financial relationships that could be construed as a potential conflict of interest.
